# Cases of Acute Intestinal Obstruction Treated by Operation
1Paper read before the Bath and Bristol Branch of the British Medical Association, Jan. 31st, 1900.


**Published:** 1900-06

**Authors:** W. H. Harsant

**Affiliations:** Surgeon to the Bristol Royal Infirmary


### CASES OF ACUTE INTESTINAL OBSTRUCTION TREATED BY OPERATION.[1]

BY

W. H. Harsant, F.R.C.S.,

*Surgeon to the Bristol Royal Infirmary.*

There are probably no cases which cause more anxiety to the surgeon than cases of acute intestinal obstruction. "A young man in robust health is seized with symptoms of strangulation in the early morning, and before the night has come an operation is advised, which is acknowledged to be attended with an enormous risk to life." "Operation in these cases is too often regarded as a last resource. It should be regarded as the *first* resource, as it certainly is the *only* resource." (Treves.)[2]

It must always be the case that a large proportion of these operations will prove fatal. A certain number will prove successful; and as during the last twelve months I have been

[1] Paper read before the Bath and Bristol Branch of the British Medical Association, Jan. 31st, 1900.

[2] *Intestinal Obstruction*, 2nd Ed., 1899, pp. 476, 477.

fortunate enough to have two recoveries out of three operations, I have felt justified in bringing them forward to encourage others in operating upon similar cases with hope of similar success.

### Case I.—Acute obstruction of the small intestine by an impacted gallstone. Operation. Recovery.

Mrs. V., aged 60, was quite well until midnight on Wednesday, May 17th, 1899, when she was seized, while in bed, with violent pains in the abdomen and vomiting. The pain was chiefly in the region of the umbilicus, but extended over the whole abdomen. The vomiting was frequent and severe. At first large quantities of green fluid were vomited, but the quantity gradually became less. The pain and vomiting continued for four days, during which time there was no action of the bowels, although copious water enemata were administered from time to time. There was some idea on the part of the patient that flatus was occasionally passed during this period, but there was some doubt about this. At first the case appeared to be one of hepatic colic, but it gradually developed into one of intestinal obstruction, the vomiting being slightly fæcal on the fourth day (Sunday).

The patient was under the constant observation of Dr. Barclay Baron, and was seen also by Dr. Markham Skerritt. I was asked to see her with a view to operation on Sunday night, May 21st, when the symptoms had lasted four days. I found the abdomen distended and tympanitic; no lump could be felt anywhere. Examination by the rectum revealed nothing. It was obviously a case of acute intestinal obstruction, and I thought the cause was probably an internal strangulation by band or volvulus. Having opened the abdomen by an incision in the median line, I found the small intestine extremely distended and very dark in colour. I drew out several feet of distended gut on to hot towels, and then, on passing my hand into the abdomen, I easily felt a hard mass blocking the intestine at one place. Above this the intestine was greatly distended, while below it was contracted and empty. As the lump was in the small intestine, I thought it could not be a fæcal mass; moreover, it felt too hard for this; so I then thought, as it afterwards proved, that it was a large gallstone. I made a longitudinal incision over the stone, and without much difficulty extracted it from the intestine. I then sutured the intestine with two rows of Lembert's sutures and returned it into the abdomen. I do not know the exact portion of the intestine which was obstructed, but it was high up in the canal and probably in the jejunum.

The after-progress of the case was uneventful. The vomiting ceased at once after the operation; the bowels acted after an enema the next day; nutrient injections were given for two days, and the patient was then allowed to take peptonised milk. She made a complete recovery.

With regard to the diagnosis of the cause of obstruction, there was nothing in the patient's history to indicate gallstones. She was never known to have passed any, and there was no previous history of attacks of colic or of jaundice. Everything pointed to a suddenly arising cause, such as a volvulus or some similar condition, and there was nothing to lead us to suspect the actual condition.

The stone when first removed was of an oval shape, measuring

$1\frac{5}{8}$ in. by $1\frac{1}{4}$ in. It weighed 160 grains when dry. It was of a very dark, almost black, colour on the exterior, and of a pale yellow on section. Owing to a rough exterior it could not readily be moved up or down through the intestinal walls, and I was afraid to use any force through the inflamed intestine in attempts to crush it for fear of injuring the already weakened intestinal coats.

A chemical examination of the stone showed: 1. A reaction for bile pigments when treated with nitric acid. 2. Pettenkofer's test showed biliary acids. 3. When dissolved in ether and evaporated it gave crystals of cholesterine. 4. Some magnesium phosphate was found in it. 5. No lime salts could be found. The mass, therefore, was a typical gallstone.

The occurrence of acute intestinal obstruction from an impacted gall-stone is not very uncommon, although I have never met with another case.

Osler *(Principles and Practice of Medicine)* describes forty-four cases of intestinal obstruction by foreign bodies, of which twenty-three were gallstones — eighteen were in women and five in men. In six-sevenths of the cases it occurred after the fiftieth year. The obstruction is usually in the ileo-cæcal region, but it may be in the duodenum. These large gallstones ulcerate through the gall-bladder, usually into the small intestine, occasionally into the colon. Courvoisier[1] has collected 131 cases in literature.

Leichtenstern (in Ziemssen's *Cyclopædia*)[2] says: "I agree with Habershon, Brinton, and others, in thinking that the diagnosis of obstruction by gall-stones is possible under favorable circumstances."

The appearance of symptoms of occlusion, immediately after a severe attack of colic, accompanied by jaundice, sometimes with an accurate history of previous attacks of hepatic colic, perhaps of the passage of gall-stones, or the rare occurrence that a small gall-stone is vomited during the attack, with attention to the age and sex, all aid in the diagnosis.

Leichtenstern[3] says: "The gall-stone may lodge at any point in the small intestine, most rarely in the middle of the ileum, more frequently in the duodenum and jejunum, most frequently . . . . in the lowest part of the ileum, one or two inches above the ileo-cæcal valve." Thirty-two cases

1 Quoted by Osler, *Op. cit.*, p. 534.

2 Vol. VII., 1877, 625. 3 *Ibid*, p. 574.

were females, nine males. It nearly always occurs late in life, after the age of 50, and most frequently after 60.

Treves, in his work on *Intestinal Obstruction*, gives a good account of this affection, and alludes to a table of 139 cases collected by Schüller.

Maylard, in his work on *The Surgery of the Alimentary Canal*, has tabulated ten successful cases of operation for this affection.

I next wish to describe briefly two cases of operation for acute intestinal obstruction by "a band."

### Case of acute intestinal obstruction by band. Operation. Death.

The patient was a lady, aged 55 years, whom I was asked to see one day last year by Dr. Walker Dunbar. Her symptoms commenced very suddenly, with great pain, coming on after a stool (the result of a dose of cascara sagrada). The pain was all over the abdomen, but worse in the region of the umbilicus. There was no vomiting the first day, and scarcely any the second day; but on the third day vomiting became frequent, and on this day I opened the abdomen. Having drawn out some coils of distended small intestine, I came upon a band of firm lymph, stretching across the abdomen and tightly constricting the gut. The band was ligatured and divided, and the operation was rapidly completed, so that it lasted less than a quarter of an hour. Within two hours of the operation there was a copious evacuation of the bowels, but this instead of relieving the patient seemed rather to increase the collapse. She never really rallied, being naturally feeble, and died about twelve hours after the operation.

The case was a great disappointment to me, as it seemed so satisfactory in every way. I attributed the failure to two causes: one being the usual one, that I operated too late, and the other being that the paralysed and distended gut above the obstruction ought to have been emptied at the time of operation to allow it to recover itself afterwards, and this I determined to effect in a subsequent case when I had the opportunity.

### Case of acute intestinal obstruction by band. Operation. Recovery.

W. H., a man, aged 50, was admitted into the Bristol Royal Infirmary on December 10th, 1899, complaining of pains in the abdomen and vomiting.

There was no history of any previous disease or illness except syphilis, for which he had been treated for some time. He might be described as of regular habits, for he assured me that he never got drunk except on Saturday nights, but that he always got fuddled with beer on that night, and in that condition went to bed on the night of Saturday, December 9th, 1899. He awoke about 3 a.m. on the morning of December 10th, with acute pain in the abdomen. He vomited several times, and the pain grew rapidly worse.

He came to the Royal Infirmary during the day and was admitted as an in-patient. The usual symptoms of acute intestinal obstruction continued until the 13th, when his condition was obviously becoming grave. The abdomen was fully distended and tympanitic in front, while in the flanks it was dull, giving rise to the diagnosis that the obstruction might be due to suppurative peritonitis, perhaps from appendicitis. There had been no passage of fæces or flatus since the commencement of the illness. I operated on December 13th, opening the abdomen by an incision in the median line. The first thing noticed on opening the peritoneum was a considerable quantity of dark fluid blood or blood-coloured serum in the peritoneal cavity, which had obviously caused the dulness in the flanks. After the fluid had escaped, the hand passed into the abdomen readily discovered a tight band connected with the lower border of the great omentum tightly strangulating the small intestine. This was ligatured and divided, and the injured portion of the intestine was brought outside and thoroughly examined. Although it had been very tightly constricted, yet I thought it would recover. The intestine above the constriction was of such a deep colour, and was so enormously distended with gas and fluid, that I determined to incise it and let out as much of the contents as I could, in order to save the patient from the toxic effects of fæcal poisoning so commonly the cause of death in these cases. I made a small incision about half an inch long in the transverse diameter of the gut, and let out about a pint of brown liquid fæces as well as a large quantity of gas. I then sutured the incision with a double row of Lembert's sutures, and restored the gut to the abdomen. The abdominal wound was closed without drainage and the operation rapidly completed.

The after-progress of the case was all that I could wish. The patient was fed entirely by the rectum for the first two days. The bowels acted for the first time on the 15th, two days after operation; he was then allowed food by the mouth, and on Christmas day, twelve days after the operation, he ate a Christmas dinner of roast beef, and sang a song after dinner. He was sent to the Convalescent Home on January 10th, four weeks after the operation.

I attribute his recovery largely to the fact that I emptied the bowel of its contents at the time of operation. Mr. Treves speaks highly of this plan, and says he considers that this addition to the operation has reduced the mortality by 50 per cent. Mr. Greig Smith always maintained that this was an important procedure, and strongly recommended its adoption. I shall not hesitate in future to make use of it in all similar cases.

